# A Novel Newborn Screening Program for Sickle Cell Disease in Nigeria

**DOI:** 10.3390/ijns10040067

**Published:** 2024-09-30

**Authors:** Aisha A. Galadanci, Umma A. Ibrahim, Yvonne Carroll, Yusuf D. Jobbi, Zubaida L. Farouk, Aisha Mukaddas, Nafiu Hussaini, Bilya Sani Musa, Lauren J. Klein, Michael R. DeBaun

**Affiliations:** 1 Department of Hematology, Bayero University/Aminu Kano Teaching Hospital, Kano 700233, Nigeria; amalgala272@gmail.com (A.A.G.); aisha1987.as@gmail.com (A.M.); 2 Department of Pediatrics, Bayero University/Aminu Kano Teaching Hospital, Kano 700233, Nigeria; aummaibraheem@gmail.com (U.A.I.); faroukzubaida@yahoo.com (Z.L.F.); 3 Department of Hematology, St Jude Children’s Research Hospital, Memphis, TN 38105, USA; yvonne.carroll@stjude.org; 4 Department of Mathematical Sciences, Bayero University Kano, Kano 700233, Nigeria; nafiu_hussaini@yahoo.com; 5 Department of Administration, Aminu Kano Teaching Hospital, Kano 700233, Nigeria; bilyasani@yahoo.com; 6 D. Brent Polk Division of Pediatric Gastroenterology, Department of Pediatrics, Hepatology, and Nutrition, Monroe Carell Jr. Children’s Hospital at Vanderbilt, Nashville, TN 37232, USA; lauren.klein@vumc.org; 7 Vanderbilt Institute for Global Health, Nashville, TN 37232, USA; 8 Department of Pediatrics Vanderbilt-Meharry Center of Excellence in Sickle Cell Disease, Vanderbilt University Medical Center, Nashville, TN 37232, USA

**Keywords:** newborn screening, genetic counseling, Africa, sickle cell disease

## Abstract

Newborn screening for sickle cell disease (SCD) is sparse in sub-Saharan Africa. The leadership of the Aminu Kano Teaching Hospital (AKTH) in Kano, Nigeria, with the support of local religious authorities, established a groundbreaking SCD newborn screening program that has become the standard of care for pregnant women and their newborns. Our program includes (1) prenatal genetic counseling for all pregnant women in the antenatal clinic, (2) newborn screening, (3) postnatal genetic counseling for parents of newborns diagnosed with SCD and SCT, and (4) referral of newborns with SCD for follow-up in the SCD Comprehensive Care Clinic by 3 months of age. From September 2020 to December 2023, the team screened 7530 infants for SCD at the AKTH, identifying 126 (1.7%) infants with SCD and 1546 (20.5%) with SCT. Of these, 93 (73.8%) newborns with SCD received individualized genetic counseling, and 43 (46%) were referred to the SCD Comprehensive Care Clinic before 3 months. Group genetic counseling was provided to the parents of 778 (50.3%) of newborns identified with SCT. The SCD newborn screening at the AKTH is now standard care, indicating the viability of sustaining an SCD newborn screening program that provides pre- and postnatal genetic counseling and comprehensive SCD care within a low-income setting.

## 1. Introduction

Sickle cell disease (SCD) is one of the most common inherited disorder worldwide [1,2] characterized by the presence of Hb S, from either homozygosity (Hb S/S) or compound heterozygosity, with another β globin variant (e.g., S/β0-thalassemia, S/βþ-thalassemia, S/O-Arab, S/D-Punjab, S/C disease) [3]. In sub-Saharan Africa, SCD is a major public health concern. The prevalence of SCD in newborn populations in low-middle-income countries, such as Nigeria, remains high, highlighting the need for further progress in early diagnosis in these regions [4,5]. A systematic analysis of the burden of SCD over 21 years from 2000 to 2021 revealed an annual increase in the incidence of SCD by 13.7% to 515,000 cases in 2021.The number children and adults with sickle cell disease globally increase 41% from 5.46 million in 2000 to 7.74 million in 2021 [6]. In the United States and other high-income countries, SCD newborn screening, parental anticipatory guidance, and age-specific vaccines have increased survival in children with SCD to approximately 98% [7,8,9]. In contrast to high-income countries, SCD-related childhood mortality in sub-Saharan Africa remains high at 50–90% [10], with fewer than half of the children reaching their fifth birthday. The major causes of death in children under 5 years of age with SCD include diarrhea, pneumonia, malnutrition, malaria and other infections, severe anemia, and stroke [11,12,13,14,15,16,17,18,19]. The major causes of death in children under 5 years of age with SCD include invasive bacterial infection and sepsis from encapsulated organisms (especially pneumococcus) [20], splenic sequestration and malaria [21], and strokes [19].

The screening of newborns for SCD allows for the early initiation of prophylactic penicillin therapy, parental education, and comprehensive management, which reduces infant morbidity and mortality [22]. Numerous efforts to implement SCD newborn screening in sub-Saharan Africa have shown initial success but lacked sustainability. However, recent funding sources provide new opportunities to improve the health of infants with SCD [23,24,25] under the comment section. 

Despite the significant prevalence of children born with SCD in Nigeria, there is no comprehensive statewide or national newborn screening program that encompasses prenatal SCD counseling, along with subsequent genetic counseling for parents of newborns with sickle cell trait (SCT) and SCD, with referral to a SCD Comprehensive Care Clinic that includes pediatric hematologists, parental anticipatory guidelines, access to hydroxyurea healthcare providers with appropriate medical expertise, and transcranial Doppler screening. The vast majority of children present with a first SCD-related complication (will now be reference, dactylitis, stroke, splenic sequestration, or sepsis), which frequently results in death [10]. 

We tested the hypothesis that a novel, sustainable, multi-modal, maternal–newborn SCD screening program could be established in Kano, Nigeria, that includes (1) prenatal genetic counseling for all pregnant women in the antenatal clinic, (2) postnatal genetic counseling for parents of newborns diagnosed with SCD and SCT, and (3) referral of newborns with SCD for follow-up in the SCD Comprehensive Care Clinic by 3 months of age.

## 2. Materials and Methods

### 2.1. Study Design and Participants

This longitudinal study was conducted at the Aminu Kano Teaching Hospital (AKTH) in Kano, Nigeria’s second-largest city, with an estimated population of 15 million and an estimated current metro area population of 4,491,000, in 2024, a 3.29% increase from 2023 [26]. The AKTH is a 700-bed tertiary-level facility. The study was approved by the Ethics Committee of the AKTH on 30 May 2018 (NHREC/21/08/2008/AKTH/EC/2235). The legal guardians of the newborns provided written informed consent before the start of the study. Dry bloodspot samples were collected from consented newborns between September 2020 and December 2023, and HPLC validation commenced in September 2019 (Table A1). We also received Ethics Committee approval for the retrospective cohort evaluation of children who received newborn screening and follow-up as standard care at the AKTH for infants beyond 28 days of life (NHREC/28/01/2020/AKTH/EC/3815, on 11 June 2024). 

### 2.2. Purchasing and Validation of the HPLC Machine

Samples from infants at the AKTH were tested for SCD using The Variant Newborn Screening (Vnbs) high-performance liquid chromatography (HPLC) system manufactured by Bio-Rad Laboratories, Hercules, CA, USA. Acquired and installed in 2019, the validation of the HPLC machine involved analyzing blood samples from individuals with known SCD, which were subsequently confirmed by the newborn screening reference laboratory at the Noguchi Memorial Institute for Medical Research, College of the Health Sciences, University of Ghana, using isoelectric focusing.

### 2.3. Training of Laboratory Technicians

Training materials and standard operating procedures were created for laboratory scientists and technicians. These materials were utilized to instruct them on sample collection, screening methods, technical operations of the HPLC machine, the newborn screening bloodspot card, puncher interface, and result interpretation. In-house training was provided by the suppliers of the HPLC machine, Pinecrest Limited, Lagos, Nigeria.

### 2.4. Quality Control

To ensure the accuracy of the newborn screening results, a suitability test was performed before each analysis to confirm the system’s integrity. The accuracy and precision of the HPLC machine were routinely assessed by examining control samples. These samples were derived from a known concentration stock solution and were analyzed alongside patient samples. Around 250–300 samples were processed monthly in two-week batches. During initial validation, approximately 15% of the newborn screening samples underwent repeat testing, comprising roughly 50 monthly samples. Repeat tests were performed for indeterminate samples (screen patterns that were inconclusive, i.e., possible contamination or readings of FAS/FSA). Confirmatory samples were obtained for indeterminant results, and these samples underwent independent confirmation and validation. Less than 1% of the samples were indeterminant (*N* = 57).

### 2.5. Sample Collection, Procedure, and Storage

Dry bloodspot samples were collected at the AKTH from newborns in the Labor Ward, Special Care Baby Unit, Postnatal Unit, and the Pediatric and Immunization Clinics. Using a Lancet Litetouch Sterile blood lancet, manufactured by Medicare, Nashville, TN, the heel prick procedure was performed to fill five bloodspots on an Ahlarstrom Munksjo screening card (Ahlarstrom Munksjo TFN). Each card was distinctly marked with a unique identification number utilized for subsequent laboratory testing by HPLC.

Stringent post-collection precautions were taken to ensure the integrity of the samples. They were subjected to a careful drying process to safeguard against exposure to sunlight and potential insect interference. The samples were placed within individual polythene bags to prevent contamination during the processing phase. Maintaining separation, the samples were stored within a temperature range of 3–8 °C until they were ready for processing via the HPLC machine. This processing occurred within a time frame of 78 h from the initial collection.

The study gathered sociodemographic data, encompassing parental names; phone numbers; home addresses; next of kin or secondary phone numbers; and details about the mother, such as age at birth, level of education, and occupation. Additionally, information on newborns, including gender, birth weight, height, and age, was collected at screening. These details were recorded in a physical case file and obtained with proper consent in an electronic database.

### 2.6. Sample Processing Using HPLC 

The Bio-Rad Vnbs HPLC machine used in this study is recognized as a standard technique in automated newborn screening for SCD and other hemoglobin disorders. The machine features a fully automated analysis of hemoglobin eluates and provides advanced result reports for maximum efficiency and clinical use, using dry bloodspot specimens, whole-blood primers, and retention time markers controls 1 (FAES) and 2 (FADC). HPLC was used to identify the most clinically significant hemoglobin variants, including HbS; HbC; HbD; HbE; and a normal hemoglobin HbA, HbF, and HbA2, as for abnormal hemoglobin variants in the dry bloodspot samples using the Genomic Data Management Software Biorad GDM 3.2 version within 3 min run time. 

### 2.7. Result Reporting

Infants were screened at the Labor Ward (within hours after birth), Postnatal Ward (up to 24 h after birth), Special Care Baby Unit (shortly after birth until discharge), Immunization Clinic (24 h and above), and the Pediatric Clinic (6 weeks and beyond). A hematologist experienced in hemoglobinopathy diagnosis interpreted the results.

The indeterminate samples were interpreted based on the S/A ratio and family history. In addition, the HPLC or POC test was repeated at 6 months or older. Any participant with an S/A ratio of >1.5 is likely SCD, while an S/A ratio < 1.5 is SCT. Family history was also taken to determine the phenotype of the parents and ascertain if they have siblings with SCD. The Bio-Rad machine also provides a pattern of FSa, which is distinguished from FSA. FSa samples the little FSa are interpreted as contamination. 

**Figure 1:**
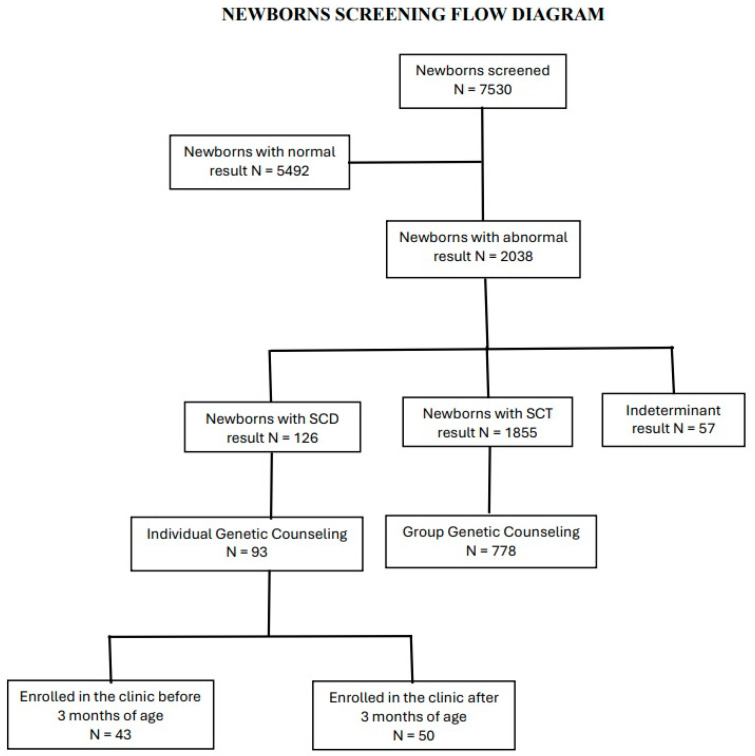
The flow diagram of newborn screening at AKTH, excluding the indeterminate samples because we had not yet understood the clinical relevance of these samples (*N* = 57).

If the results were positive for SCD, mothers were informed and promptly scheduled for an appointment in the SCD Comprehensive Care Clinic, held every Friday. If the results were positive for SCT, mothers were notified and scheduled for the next monthly group genetic counseling session (Figure 1). During study enrollment, a stable secondary contact person, such as a grandparent, neighbor, teacher, or friend, was identified to assist in reaching the mothers. In cases where the mother was unavailable, we reached out to the secondary contact person to ensure the successful communication of the results. 

### 2.8. Genetic Counseling Training and Process

Parents of newborns with SCD received individualized genetic counseling during their initial visit to the SCD Comprehensive Care Clinic from the AKTH nurses. The nurses received training from The Comprehensive Sickle Cell Center at the University of Cincinnati and Cincinnati Children’s Hospital. The nurses participated in a two-day online hemoglobinopathy counselor training course on 14–15 April 2021. Some course content included information about SCD genetics, pathophysiology, and psychosocial and medical issues related to SCD and other hemoglobinopathies. 

The nurses provided group genetic counseling for parents of children with SCT on the third Saturday of each month. During the sessions, AKTH nurses discussed genetic factors associated with SCD; explained the inheritance pattern of SCD; and provided information on SCT and SCD, including symptoms, treatment, and prevention. Additionally, for parents of newborns with SCD, nurses emphasized the significance of early and ongoing enrollment in the SCD Comprehensive Care Clinic.

### 2.9. Enrollment in the SCD Comprehensive Care Clinic

Infants diagnosed with SCD through newborn screening were referred to the SCD Comprehensive Care Clinic. The target age for enrollment was less than 3 months old. Enrollment before 3 months of age facilitates the initiation of parental anticipatory guidance [22] and the commencement of penicillin prophylaxis to prevent streptococcal pneumonia infection [22,23,24,25,26,27]. The established public health strategies have been shown to reduce infant mortality. Comprehensive medical care included but was not limited to the screening and treatment of the five major causes of death in children under five years of age living in Nigeria, vaccinations to prevent life-threatening bacterial infections, insecticide-treated malaria nets and anti-malaria prophylaxis, screening and treatment for severe acute malnutrition, screening and treatment for acute chest syndrome, and screening and treatment for diarrhea [28,29,30,31,32]. The SCD Comprehensive Care Clinic also provided specific SCD anticipatory guidance and primary prevention, daily penicillin prophylaxis, transcranial Doppler screening for primary prevention of strokes, and treatment with hydroxyurea for stroke prevention if the TCD velocities aretelevated [33]. Simillary secondary stroke prevention with hydroxyurea for the children with sickle cell anemia [34].

### 2.10. Integration of Newborn Screening into the Antenatal Care Package with the Involvement of Community Health Workers (CHWs)

In 2023, three years following the commencement of the newborn screening project, AKTH management incorporated SCD newborn screening as a part of their antenatal package. This package includes prenatal SCD genetic counseling for all pregnant women treated in the clinic, pre- and postnatal counseling for women with SCT and SCD, and HPLC newborn screening for SCD. Currently, every newborn delivered at the AKTH undergoes SCD testing.

At the AKTH, prenatal education is routinely provided to pregnant women in the waiting room during antenatal clinic visits. Beginning in May 2023, SCD health education was added to the prenatal education protocol. Two CHWs provide anticipatory education about having a child with SCD. This counseling is provided to all pregnant women during the prenatal clinics held four days a week, from Monday to Thursday. The CHWs were formally trained to conduct education sessions describing SCT and the inheritance pattern of SCD. Additionally, if a pregnant woman has SCT or SCD, the CHWs conduct individual meetings with them during the third trimester to underscore the importance of seeking timely follow-up care if their child is diagnosed with SCD. During prenatal visits, early education on SCD helps expectant mothers understand the condition, its implications, and the importance of early intervention.

After reassessing the percentage of families with newborns with SCD enrolled in the SCD Comprehensive Care Clinic, the roles of the CHWs were expanded to include home visits to increase enrollment of newborns in the clinic before 3 months of age. 

### 2.11. Statistical Analysis

Categorical variables were summarized using frequency and percentages; continuous variables were summarized as means with standard deviations and medians and interquartile ranges when not normally distributed. Early enrollment into the SCD Comprehensive Care Clinic was defined as before 3 months of age. Maternal sociodemographic factors associated with early enrollment were examined using the chi-square test (for occupation) and Fisher’s exact test (for educational level). The Mann–Whitney test was used to compare maternal age between the two groups. All statistical analyses were performed in STATA (Statistical Software, Release 15, StataCorp 2017, https://www.stata.com/stata15/).

## 3. Results

### 3.1. Demographic Characteristics of Study Participants

This study involved a total of 7530 newborns delivered at the AKTH between September 2020 and December 2023. The average weekly delivery at the AKTH is 70–80 births. The demographic characteristics of newborns screened are described in Table 1. Females accounted for 51.5% of the newborns. The median weight was 3.0 kg, with an interquartile range of 2.7–3.5 kg. Approximately half of the newborns were screened on the first day of delivery (median = 1.0 days, IQR < 1.0–6.0 days). Infants were screened in 2020, 2021, 2022, and 2023. Bloodspot samples were collected from newborns and infants in the following areas: 46.4% (3494 of 7530) from the Labor Ward, 35.3% (2660 of 7530) from the Immunization Clinic, 16.5% (1240 of 7530) from the Special Care Baby Unit, 1.6% (120 of 7530) from the Postnatal Unit, and 0.2% (17 of 7530) from the Pediatric Clinic. 

### 3.2. HPLC Results

**Figure 2:**
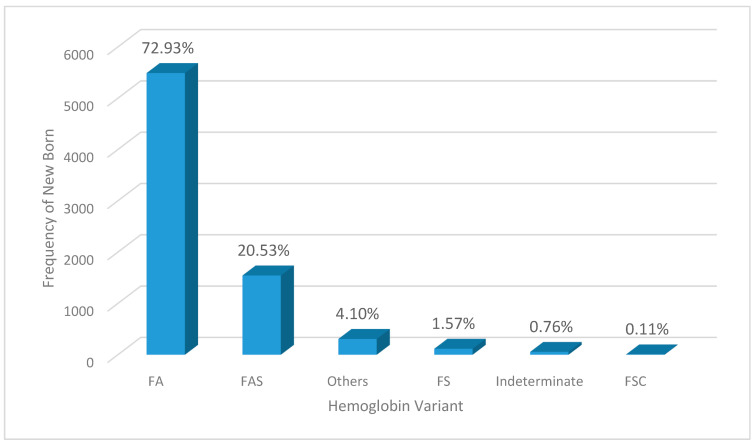
Pattern and distribution of hemoglobin variants for newborns in the AKTH obtained using HPLC from September 2020 to December 2023 (*n* = 7530).

The hemoglobin phenotype patterns were Hb FA 72.9% (*n* = 5492) and FAS 20.5% (*n* = 1546), followed by the predominant SCD phenotypes of Hb FS 1.6% (*n* = 118) and FSC < 0.1% (*n* = 8). For 4.1% of the samples (*n* = 309), there were other hemoglobin variants, i.e., FAC, FAD, FAE, and 0.8% (*n* = 57) were indeterminant samples (inconclusive or FSA/FAS/FSa) (Figure 2). 

Of the 57 indeterminate samples, 10.5% (*n* = 6) had FSa, and 89.5% (*n* = 51) had FSA. 

HPLC was repeated at 6 months or older for 83.3% (*n* = 5) of the six indeterminate samples with FSa, and all 100% (*n* = 5) had a S/A ratio of >1.5 and phenotype of SS following the retest. Only one participant with FSa did not have a retest because the family could not be contacted as their phone number was switched off, but he had a S/A ratio of >1.5, suggesting a likely diagnosis of SCD instead of SCT. 

Among the 51 indeterminate samples with FSA, only 37.3% (*n* = 19) had a retest, whereas 62,7% (*n* = 32) did not retest because the parents could not be contacted via phone.

Of the 19 participants with FSA who had a retest, 68.4% (*n* = 13) had a POC testing after the HLPC, while 6 (31.6%) had HPLC. All 100% (*n* = 19) of the participants had a phenotype of AS. All 100% (*n* = 19) of the participants with FSA who had a retest had a S/A ratio of <1.5, and the parents of 84.2% of the newborns (*n* = 16) had sickle cell traits, while 15.8% (*n* = 3) did not know their phenotype. The S/A ratio was calculated for the 32 indeterminate samples with FSA that could not be retested, and the majority had a S/A ratio of <1.5, so they most likely had SCT. 

### 3.3. Genetic Counseling Results 

Individual genetic counseling was provided for parents of newborns with SCD, and group counseling was provided for those with SCT. Notably, 93 (73.8%) of parents of newborns with SCD received individual counseling, while 778 (50.3%) of parents of newborns with SCT received group counseling.

### 3.4. Follow-Up of Newborns in the SCD Comprehensive Care Clinic

A total of 93 (73.8%) newborns with SCD identified by newborn screening at the AKTH between September 2020 and December 2023 were enrolled in the SCD Comprehensive Care Clinic. Forty-three (46.2%) of those newborns were enrolled before 3 months of age, started on penicillin and antimalarial prophylaxis, and received anticipatory guidance for splenic sequestration detection, fever management, and other preventive strategies. The age of enrollment of the remaining 50 newborns enrolled in the SCD Comprehensive Care Clinic ranged from 3.2 months to 14.3 months, with a mean of 7.4 and a median of 6.4 months (IQR 4.7–10.6). 

## 4. Discussion

We have implemented one of the first sustainable integrated maternal–newborn SCD screening programs in Nigeria. A unique aspect of our program is the addition of SCD newborn screening as standard clinical care for all newborns at the AKTH; (1) prenatal SCD genetic counseling for all pregnant women in the antenatal clinic, (2) postnatal genetic counseling for parents of newborns diagnosed with SCD and SCT, and (3) referral of newborns with SCD for follow-up in the SCD Comprehensive Care Clinic before 3 months of age.

During the development of the multi-modal program, new strategies were implemented to gain community acceptance, increase reproductive decision-making, and decrease SCD-related morbidity in the first five years of life through establishing an SCD Comprehensive Care Clinic. Kano is the second largest city in Nigeria, with a mostly Muslim population adhering to a strict religious hierarchy. Patient-oriented studies must incorporate the cultural diversity and multiplicity of the community. To respect cultural values, we sought support from the emir of Kano and the religious leaders of various Islamic sects. We met with the Imams (mosque prayer leaders) individually and subsequently organized a workshop for 120 Imams in Kano to solicit support for newborn screening and genetic counseling [35]. Second, with the support of CHWs, we established a maternal SCD education program during the prenatal visits to provide education and reinforce the importance of prompt follow-up for newborns with positive SCD results. Third, after significant preparatory work to understand barriers and facilitators for genetic counseling [36]. AKTH hematologists introduced individual genetic counseling for parents of newborns with SCD and group counseling for parents of children with SCT. Lastly, we established an SCD Comprehensive Care Clinic to refer infants identified with SCD during newborn screening and provide standardized, evidence-based medical care, including transcranial Doppler screening (TCD) with hydroxyurea treatment for those with abnormal TCD velocities.

Numerous newborn screening programs have been established across Africa, with many being initiated through philanthropic or grant-funded projects [11]. Some of the earliest programs were initiated in Ghana [37], Benin [38], Angola [39], and the Democratic Republic of Congo [40]. However, to the best of our knowledge, our program distinguishes itself as the only initiative to incorporate a comprehensive SCD newborn screening program as standard practice within an institution established following the endorsement of hospital leadership and religious authorities and without the support of the state or federal government. We did receive initial supporfrom St. Jude Research Children’s Hospital for establishing the laboratory and under-0five clinic; however, the support for the newborn screening will stop in 2025, and the newborn screening program will be self-sustaining as part of the prenatal care service bundle that the hospital offers. Existing hospital personnel and equipment will be used for the continued implementation of the program, and hydroxyurea and trans-cranial Doppler, included as part of SCD comprehensive clinic care, will be provided by the hospital; however, additional hospital or government support will be required to expand the program to hospitals outside of the AKTH. 

The AKTH embraced and expanded the SCD newborn screening program by integrating SCD education in the prenatal clinic and providing additional personnel (CHWs) dedicated to SCD genetic counseling. Furthermore, AKTH leadership has provided the services of two extra CHWs to assist in conducting home visits and facilitate follow-up for newborn screening. The AKTH has implemented the entire newborn screening program as part of its standard care protocol, which includes (1) prenatal genetic counseling for all pregnant women in the antenatal clinic, (2) SCD newborn screening, (3) postnatal genetic counseling for parents of newborns diagnosed with SCD and SCT, and (4) referral of newborns with SCD for follow-up in the SCD Comprehensive Care Clinic by 3 months of age. All four elements are essential for an SCD newborn screening program. A significant challenge of implementing a newborn screening program in a low-resource setting is the cost and sustainability beyond the initial funding. These include the overall costs associated with implementing the program, acquiring the necessary equipment and consumables, and adequately training healthcare personnel. The cost of HPLC per individual is USD 8.00. Comparatively, per person, sickle-scan point-of-care testing costs USD 3.00–4.00. These costs are prohibitive to most families in Nigeria due to endemic poverty, where families pay out of pocket for healthcare services. Regardless of the SCD newborn screening technique, the laboratory tests are not affordable for 30.9% of the Nigerian population, who live on approximately USD 2.15 0 [41]. This highlights the need for comprehensive policies and sustainable funding to implement newborn screening at the state and federal levels. Importantly, based on the success of the newborn program SCD screening program and the support from religious leaders, the state has implemented a premarital genetic testing requirement for SCD and other diseases. Furthermore, buy-in by hospital management is a must; at the AKTH, it led to integrating the SCD newborn screening program into the routine prenatal care package. This study demonstrates local stakeholders’ critical role in championing SCD programs to ensure sustainability.

Our study had several limitations. We only obtained institutional support to initiate newborn screening for SCD at the AKTH after 3 years of philanthropy support demonstrating the added value of SCD newborn screening. Another study limitation is that only 46% of the mothers of infants with SCD returned to be evaluated within the first 3 months to start penicillin prophylaxis. While the follow up rate withn 3 months is moderate, approximately, 50% there are few comparative articles with follow-up data for a new SCD newborn screening program in a low-middle-income countries with referral of the newbnorn to a comprehensive SCD clinic for newborns by 3 months of age [42,43]. Similarlarly in the United States timely referral to a comprehensive SCD clinic is chalenging even in the United States [44,45]. To address this limitation, CHWs started attending the prenatal clinic to educate mothers with SCT or SCD about the possibility of having a child with SCD. They emphasized the importance of prompt follow-up in the SCD Comprehensive Care Clinic if their child was diagnosed with SCD. The CHWs began conducting home visits for unresponsive mothers. 

We recognize a significant limitation is the inability to perform confirmatory testing in all presumptive SCD diagnoses. Our inability to have confirmatory testing is directly related to budget restraints. The inability to perform confirmatory testing resulted in 0.8% indeterminate results. Only an extensive follow-up allowed us to capture a small portion of newborns that had intermediate NBS screening, which did not allow the providers to include or exclude a diagnosis of SCD. To overcome this limitation, we formally evaluated point-of-care testing as the initial result with follow-up HPLC evaluation for those positive for SCD or indeterminant values. Thus, all children with positive point-of-care testing would be referred to the SCD Comprehensive Care Clinic, started on penicillin, and evaluated by a pediatric hematologist for SCD for a definitive diagnosis based on HPLC testing and laboratory findings.

To our knowledge, we have described a novel newborn screening program in sub-Saharan Africa that includes prenatal SCD genetic counseling for all pregnant women in the antenatal clinic, postnatal genetic counseling for parents of newborns diagnosed with SCD and SCT, and referral of newborns with SCD to the SCD Comprehensive Care Clinic before 3 months of age for parental anticipatory guidance and medical management.

## Figures and Tables

**Table 1:** Demographic characteristics of newborns screened for SCD at the Aminu Kano Teaching Hospital Between September 2020 and December 2023.

Variables	Total Cohort (*N* = 7530)
Gender, n (%)	
Female	3880 (51.5)
Age group, n (%)	
<6 weeks	7099 (94.3)
6 weeks–3 months	316 (4.2)
3 months–<6 months	63 (0.8)
≥6 months	53 (0.7)
Weight, kg	
Median (IQR)	3 (2.7–3.5)
Height, cm	
Mean ± SD	48.6 ± 4.1
Age at screening (in days)	
Median (IQR)	1 (<1.0–6.0)
Year of screening, n (%)	
2020	498 (6.6)
2021	2590 (34.4)
2022	2923 (38.8)
2023	1519 (20.2)

IQR: interquartile range, SD: standard deviation.

## Data Availability

The original contributions presented in the study are included in the article, further inquiries can be directed to the corresponding author.

## Appendix A

**Table A1:** Timeline for newborn screening program in AKTH.

	Year 1 2019				Year 2 2020				Year 3 2021	Year 4 2022	Year 5 2023
	1	2	3	4	1	2	3	4			
Ethical approval	X										
Purchase of HPLC machine by St. Jude	X										
Community/health personal education and enlightenment	X										
Focus group discussions	X	X	X	X	X	X	X	X			
Validation of HPLC machine	X	X	X	X	X	X	X	X	X	X	X
Visit to the Emir of Kano State Government				X							
Visit to the Kano State Government				X							
Newborn screening							X	X	X	X	X
Informed consent and enrollment into comprehensive care as part of standard care (no research procedures)				X	X	X	X	X	X	X	X
Group and individual genetic counseling								X	X	X	X
Workshop for religious leaders on KAP of Premarital counseling for SCD			X								
Pneumococcal Immunization								X	X	X	X
Penicillin prophylaxis								X	X	X	X
An antimalarial prophylaxis								X	X	X	X
TCD (at 2 years of age)										X	X
Involvement of CHWs in routine prenatal visits											X
Including newborn screening as part of the antenatal care package											X
